# Task Failure during Exercise to Exhaustion in Normoxia and Hypoxia Is Due to Reduced Muscle Activation Caused by Central Mechanisms While Muscle Metaboreflex Does Not Limit Performance

**DOI:** 10.3389/fphys.2015.00414

**Published:** 2016-01-11

**Authors:** Rafael Torres-Peralta, David Morales-Alamo, Miriam González-Izal, José Losa-Reyna, Ismael Pérez-Suárez, Mikel Izquierdo, José A. L. Calbet

**Affiliations:** ^1^Department of Physical Education, University of Las Palmas de Gran CanariaLas Palmas de Gran Canaria, Spain; ^2^Research Institute of Biomedical and Health Sciences (IUIBS)Las Palmas de Gran Canaria, Spain; ^3^Department of Health Sciences, Public University of NavarraTudela, Spain

**Keywords:** electromyography, EMG, exhaustion, fatigue, high-intensity, hypoxia, lactate, performance

## Abstract

To determine whether task failure during incremental exercise to exhaustion (IE) is principally due to reduced neural drive and increased metaboreflex activation eleven men (22 ± 2 years) performed a 10 s control isokinetic sprint (IS; 80 rpm) after a short warm-up. This was immediately followed by an IE in normoxia (Nx, P_I_O_2_:143 mmHg) and hypoxia (Hyp, P_I_O_2_:73 mmHg) in random order, separated by a 120 min resting period. At exhaustion, the circulation of both legs was occluded instantaneously (300 mmHg) during 10 or 60 s to impede recovery and increase metaboreflex activation. This was immediately followed by an IS with open circulation. Electromyographic recordings were obtained from the *vastus medialis* and *lateralis*. Muscle biopsies and blood gases were obtained in separate experiments. During the last 10 s of the IE, pulmonary ventilation, VO_2_, power output and muscle activation were lower in hypoxia than in normoxia, while pedaling rate was similar. Compared to the control sprint, performance (IS-Wpeak) was reduced to a greater extent after the IE-Nx (11% lower *P* < 0.05) than IE-Hyp. The root mean square (EMG_RMS_) was reduced by 38 and 27% during IS performed after IE-Nx and IE-Hyp, respectively (Nx vs. Hyp: *P* < 0.05). Post-ischemia IS-EMG_RMS_ values were higher than during the last 10 s of IE. Sprint exercise mean (IS-MPF) and median (IS-MdPF) power frequencies, and burst duration, were more reduced after IE-Nx than IE-Hyp (*P* < 0.05). Despite increased muscle lactate accumulation, acidification, and metaboreflex activation from 10 to 60 s of ischemia, IS-Wmean (+23%) and burst duration (+10%) increased, while IS-EMG_RMS_ decreased (−24%, *P* < 0.05), with IS-MPF and IS-MdPF remaining unchanged. In conclusion, close to task failure, muscle activation is lower in hypoxia than in normoxia. Task failure is predominantly caused by central mechanisms, which recover to great extent within 1 min even when the legs remain ischemic. There is dissociation between the recovery of EMG_RMS_ and performance. The reduction of surface electromyogram MPF, MdPF and burst duration due to fatigue is associated but not caused by muscle acidification and lactate accumulation. Despite metaboreflex stimulation, muscle activation and power output recovers partly in ischemia indicating that metaboreflex activation has a minor impact on sprint performance.

## Introduction

Muscle fatigue has been defined as “any exercise-induced reduction in the ability to exert muscle force or power, regardless of whether the task can be sustained (Bigland-Ritchie and Woods, 1984), that can be reversed by rest” (Gandevia, 2001). The mechanisms leading to task failure may involve physiological processes at neural (central fatigue) or muscular levels (peripheral fatigue), with failure distal to the neuromuscular junction included in the “peripheral” component. It has been suggested that the rate of muscle fatigue development is regulated by the central nervous system (CNS) with feedback from the type III and IV muscle afferents (Amann and Dempsey, 2008), which sense metabolite accumulation, particularly H^+^, lactate, and ATP (Light et al., 2008). Type III/IV muscle afferents have been reported to inhibit corticospinal drive (Amann and Dempsey, 2008; Rossman et al., 2012; Kennedy et al., 2015), with greater inhibitory effect on extensor than flexor muscles (Martin et al., 2006). However, whether metabolite accumulation and the expected metaboreflex stimulation impair muscle activation and limit peak power during whole-body sprint exercise remains unknown.

During whole-body exercise in severe acute hypoxia (F_I_O_2_ < 0.115, or altitude above 4500 m) the level of peripheral fatigue at exhaustion seems lower, indicating that central mechanisms, likely linked to reduced brain oxygenation (Rasmussen et al., 2010), predominate over local mechanisms in determining the cessation of exercise (Amann et al., 2007). In agreement with this hypothesis, an instantaneous increase of the inspired O_2_ fraction (F_I_O_2_) from 0.21 to 1.00 does not eliminate muscle fatigue at the end of an incremental exercise test performed at sea level (Calbet et al., 2003a). In contrast, during constant-intensity or incremental exercise to exhaustion in severe hypoxia (P_I_O_2_ ≈ 75 mmHg), muscle fatigue is swiftly relieved by mild hyperoxic gas or room air (Kayser et al., 1994; Calbet et al., 2003a,b; Amann et al., 2007). These findings led to the concept that during incremental exercise to exhaustion in normoxia, task failure is most likely caused by peripheral mechanisms while central mechanisms prevail in severe hypoxia (Calbet et al., 2003a; Amann et al., 2007). In support, peripheral fatigue, as assessed via decreases in potentiated quadriceps twitch force 2 min after constant-intensity exercise to exhaustion, was lower when the exercise was performed in severe hypoxia than in normoxia (Amann et al., 2007). Nevertheless, this observation was not accompanied by an assessment of muscle metabolites and obviates the fact that reduced potentiated quadriceps twitch force may occur without reduction of peak power output (Fernandez-del-Olmo et al., 2013; Hureau et al., 2014). Moreover, the recovery process starts as early as muscle contraction ceases, and given the fast kinetics of phosphocreatine re-synthesis at the end of exercise (Bogdanis et al., 1996; Dawson et al., 1997; Yoshida et al., 2013), most of the recovery has already occurred within the first 2 min post-exercise (Sargeant and Dolan, 1987; Froyd et al., 2013). Also, muscle fatigue is task-specific (Gandevia, 2001), implying that a procedure using a similar pattern of movement, and hence recruitment of neural pathways, is expected to be more sensitive to detect fatigue.

In this context, every possible combination of effects and interpretation of results has been reported in regard to the contribution of central and peripheral mechanisms to muscle fatigue after dynamic contractions in normoxia (Sidhu et al., 2009, 2012; Marcora and Staiano, 2010; Fernandez-del-Olmo et al., 2013) and hypoxia (Amann et al., 2007; Goodall et al., 2010; Millet et al., 2012). Some of these discrepancies can be attributed to the fact that neural mechanisms of muscle fatigue are task-specific (Sidhu et al., 2012), to different levels of input from type III and IV muscle afferents (Sidhu et al., 2012), and to methodological limitations (Rodriguez-Falces et al., 2013; Héroux et al., 2015; Bachasson et al., 2016). Although the inhibition of type III and IV muscle afferents has been shown to attenuate muscle fatigue in certain exercise models (Sidhu et al., 2014), whether type III and IV muscle afferent input contributes to reduce exercise performance by a central mechanism remains controversial (Millet et al., 2009, 2012; Marcora, 2010; Amann et al., 2013a; Kennedy et al., 2015). Part of the discrepancies may be due to the fact that type III and IV muscle afferent discharge cannot be directly measured during whole-body exercise in humans, combined with the difficulty in interpreting the effects of intrathecal fentanyl when this drug differently alters ventilation, arterial O_2_ content, arterial PaCO_2_, heart rate and mean arterial blood pressure, depending on the exercise intensity, exercise duration, and the study population (Dempsey et al., 2014; Olson et al., 2014; Poon and Song, 2015). Thus, we decided to explore the role of III/IV muscle afferents on exercise performance (peak power output) using a completely different experimental approach.

The main aim of this investigation was to determine whether task failure during an incremental exercise to exhaustion is principally due to central mechanisms that cause a reduction in neural activation, modulated by the level of oxygenation. This hypothesis has been previously examined but with different procedures (Kayser et al., 1994; Amann et al., 2007, 2013b) which did not include measurements of muscle metabolites or performance immediately after exhaustion. Another aim was to determine if increased afferent feedback from metabolite accumulation in an exhausted muscle has a negative influence on sprint performance by reducing neural activation, as assessed through electromyogram (EMG) recordings.

We aimed to test these two hypotheses: (i) task failure during incremental exercise to exhaustion in hypoxia occurs with lower levels of muscle activation compared to normoxia, and (ii) increased afferent feedback from III and IV muscle afferents impairs sprint performance.

## Methods

### Subjects

Eleven healthy men (age: 21.5 ± 2.0 years, height: 174 ± 8 cm, body mass: 72.3 ± 9.3 kg, body fat: 16.1 ± 4.9%, VO_2_max: 51 ± 5 mL.kg^−1^.min^−1^) agreed to participate in this investigation. Before volunteering, subjects received full oral and written information about the experiments and possible risks associated with participation. Written consent was obtained from each subject. The study was performed by the Helsinki Declaration and was approved by the Ethical Committee of the University of Las Palmas de Gran Canaria (CEIH-2010-01 and CEIH-2009-01).

### General overview

This study was a part of a larger project that included several experiments designed to address the mechanisms limiting whole-body exercise performance in humans. The results focusing on O_2_ transport and muscle metabolism have been published (Calbet et al., 2015; Morales-Alamo et al., 2015). Body composition was determined by dual-energy x-ray absorptiometry (DEXA) (Hologic QDR-1500, Hologic Corp., software version 7.10, Waltham, MA) (Calbet et al., 1997), during the familiarization sessions. The leg muscle mass was calculated from the DEXA scans using Wang's et al. model (Wang et al., 1999).

**Figure 1:**
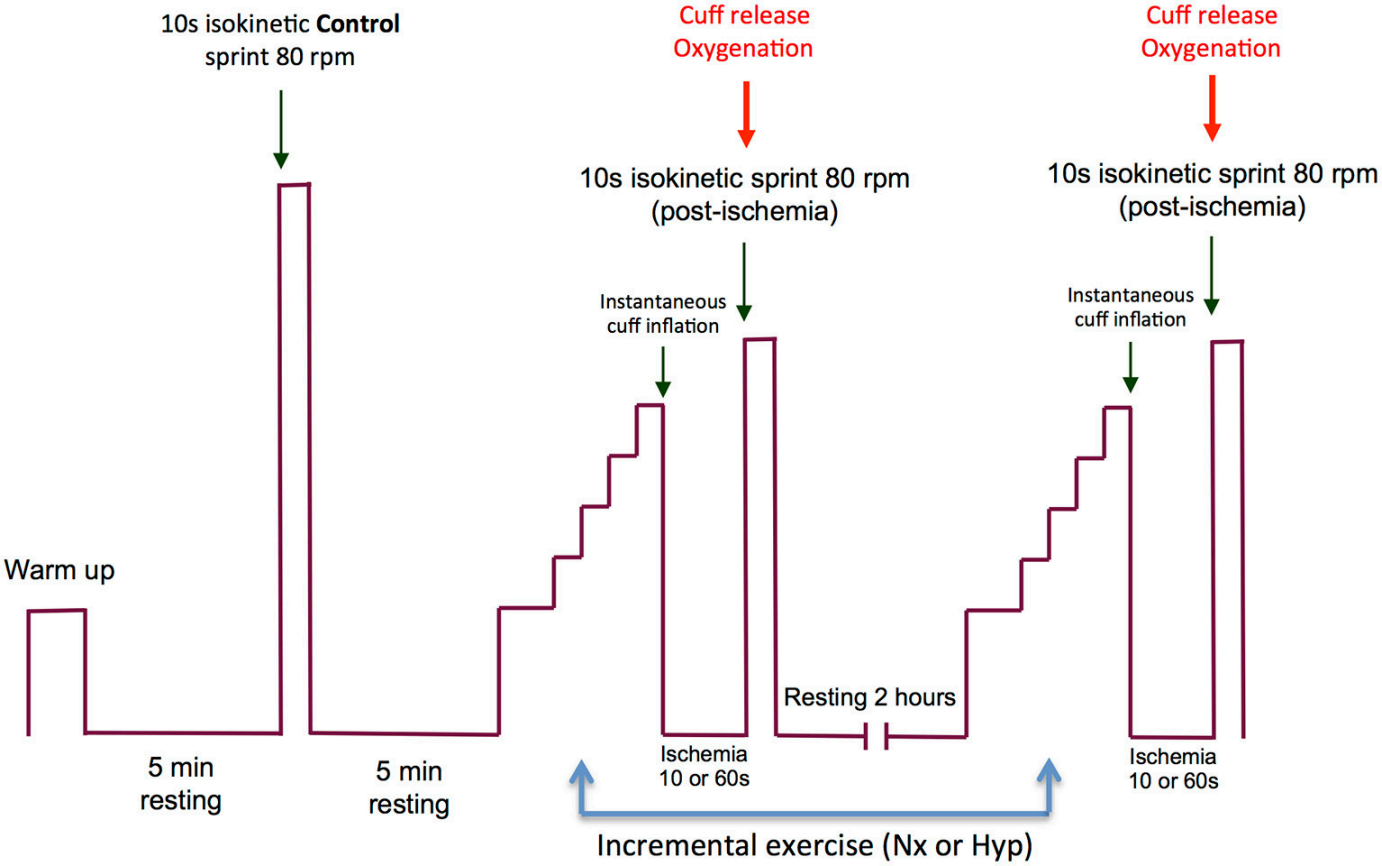
**Experimental protocol**. The experimental day started with a warm-up followed by 4.5 min of slow unloaded pedaling and a 30 s resting phase, while the subjects became ready to perform the first sprint (isokinetic, 10 s at 80 rpm) at the 5th minute after the end of the warm-up. This sprint was used as a control sprint and was always performed in normoxia. Five minutes later, an incremental exercise to exhaustion began in normoxia (P_I_O_2_: ~143 mmHg) or acute hypoxia (P_I_O_2_: ~73 mmHg). The order of the incremental exercise test was randomized. Between the two incremental exercise tests, the subjects were allowed to rest during 120 min. At the end of the incremental exercise test, bilateral cuffs were inflated at maximal speed and pressure (i.e., 300 mmHg) to occlude completely and instantaneously the circulation (ischemia) of the legs. The incremental exercise test in normoxia and hypoxia ended with an ischemia period of 10 s on one experimental day and 60 s on another day. The order of the duration of the ischemia period was randomized. At the end of the ischemia period, the subjects performed a 10 s isokinetic sprint as hard and fast as possible (80 rpm) while the cuffs were always instantaneously deflated at the beginning of the post-ischemia sprints.

**Figure 2:**
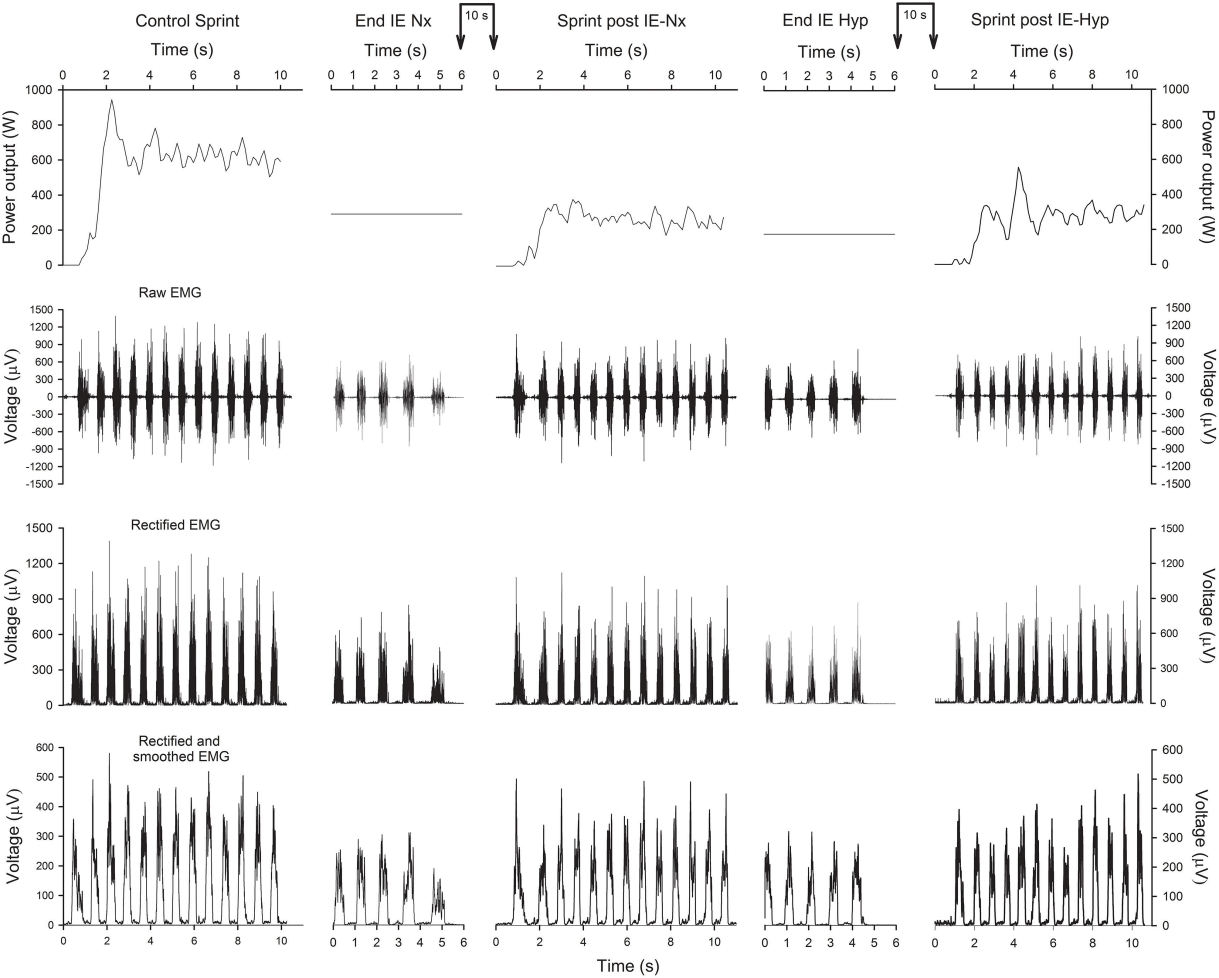
**Power output and EMG of a representative subject**. Schematic representation of the power output (upper panels), raw EMG (2nd row), rectified EMG (3rd row) and rectified and smoothed EMG (lower panels), during the control sprint, last 6 s of the incremental exercise (IE) in normoxia (Nx), subsequent 10-s isokinetic sprint at 80 rpm (normoxia), IE in hypoxia (Hyp) and subsequent 10-s isokinetic sprint at 80 rpm (normoxia). The connected vertical arrows indicate the duration of the ischemia period, which in this example was 10 s.

The experimental protocol is summarized in Figure 1, and an example of the electromyographic recordings from one subject is given in Figure 2. On the experimental days, subjects reported to the laboratory at 08.00 h. after an overnight fast from 22.00 h. The subjects performed an incremental exercise test to exhaustion in normoxia (P_I_O_2_: ~143 mmHg) or acute hypoxia (P_I_O_2_: ~73 mmHg, Altitrainer200, SMTEC, Switzerland), in random order and separated by a 120 min rest. Before the exercise test, bilateral cuffs were placed around the thighs and connected to a rapid cuff inflator (SCD10, Hokanson E20 AG101, Bellevue, USA). The test started with a warm-up (2 min at 50 W + 2 min 100 W + 1 min at 160 W) followed by 4.5 min of slow unloaded pedaling. This was followed by a 30 s rest period while the subjects became ready to sprint at the 5th minute after the end of the warm-up. The volunteers were requested to sprint as hard and fast as possible during 10 s with the ergometer set in isokinetic mode and at 80 rpm (Excalibur Sport 925900, Lode, Groningen, The Netherlands). This sprint was used as a control sprint and was always performed in normoxia. Five minutes later, the incremental exercise began. For the test in normoxia, the load was increased by 30 W every 2 min until exhaustion, starting from an initial load of 80 W. In hypoxia, the incremental test started from 60 W, and the load was increased by 20 W every 2 min until exhaustion. Exhaustion during the incremental exercise tests was defined by the subject stopping pedaling or dropping pedaling rate below 50 rpm during 5 s, despite strong verbal encouragement. At exhaustion, the cuffs were inflated at maximal speed and pressure (i.e., 300 mmHg) to completely and instantaneously occlude the circulation (ischemia). This prevented any increase of oxygenation during the recovery and caused anoxia within 3–5 s of the application of the occlusion as reported elsewhere (Morales-Alamo et al., 2015). A limitation of previous studies was that the impact of the early recovery could not be accounted for (Marcora and Staiano, 2010; Coelho et al., 2015), and certainly some recovery occurs during the time elapsed between the end of the exercise and the start of the sprint. To circumvent this limitation we applied complete ischemia during the recovery and we used short (10 s) and long (60 s) ischemia periods. We surmised that peripheral fatigue would be exacerbated by the prolonged ischemia at the end of an incremental exercise to exhaustion.

The incremental exercise test in normoxia and hypoxia ended with two different periods of ischemia of 10 or 60 s, during which the subjects breathed normoxic gas. Following a countdown, the subject performed a 10 s isokinetic sprint as hard and fast as possible while the ergometer was set at 80 rpm. The cuffs were always instantaneously deflated at the beginning of the post-ischemia sprints. In the unfatigued state, peak power increases with pedaling rate, but peak power is less affected by pedaling rate in the fatigued state (Beelen and Sargeant, 1991). Importantly, the difference in peak power between the fatigued and unfatigued state increases the higher the pedaling rate used in the control sprint (unfatigued) (Beelen and Sargeant, 1991). Thus, to avoid the limitations associated with varying pedaling rates sprints were performed in isokinetic mode at 80 rpm, a pedaling rate that allows maximal power output in the fatigued state (Beelen and Sargeant, 1991).

A few weeks later, the IEs were repeated in two additional experimental sessions; 1 day in normoxia (Nxb, P_I_O_2_: ~143 mmHg) and the other in hypoxia (Hypb, P_I_O_2_: ~73 mmHg; “b” indicates biopsy session). In the Nxb session, after 10 min rest in the supine position, a muscle biopsy was obtained from the m. *vastus lateralis* with local anesthesia (lidocaine 2%, 2 ml), using the Bergstrom technique with suction (Bergstrom, 1962). This biopsy was obtained with the needle pointing distally with 45° inclination (Guerra et al., 2011). An additional incision was performed before the beginning of the exercise in the contralateral leg. Afterward, the incisions were covered with a transient plaster, and a cuff was placed around the left leg. The subjects then sat on the cycle ergometer and resting measurements were performed. Two minutes later, the IE was begun as described above. At exhaustion, the cuff was inflated instantaneously at 300 mmHg, and a biopsy was taken exactly 10 s after the end of the incremental exercise test. The biopsy needle was introduced perpendicular to the thigh. This biopsy was followed by a final biopsy at 60 s with the needle pointing proximally (45° inclination). In the Hypb session, essentially the same procedures were applied. All biopsies were immediately frozen in liquid nitrogen and stored at −80°C. Hypb and Nxb sessions were performed in random order.

### Power output, oxygen uptake and hemoglobin oxygen saturation

Power output during the sprint was reported as instantaneous peak power output (Wpeak-i) and mean power output (Wmean) during the 10 s duration of the sprint. Oxygen uptake was measured with a metabolic cart (Vmax N29; Sensormedics, Yorba Linda, California, USA), calibrated before each test according to the manufacturer instructions, with high-grade calibration gasses (Carburos Metálicos, Las Palmas de Gran Canaria, Spain). Respiratory variables were analyzed breath-by-breath and averaged every 20 s during the incremental exercise tests. The highest 20-s averaged VO_2_ recorded in normoxia was taken as the VO_2_max. The same criterion was applied to determine the VO_2_peak in hypoxia. Hemoglobin oxygen saturation (SpO_2_) was determined with a finger pulse oximeter (OEM III module, 4549-000, Plymouth, MN).

### Muscle metabolites

From each muscle biopsy, 30 mg of wet tissue were freeze-dried, cleaned and powdered with a manual mortar on ice. Subsequently, the samples were suspended in 0.5 M HClO_4_ and centrifuged at 15,000 g at 4°C for 15 min. The supernatant was neutralized with KHCO_3_ 2.1M. ATP, phosphocreatine (PCr), creatine, pyruvate and lactate concentrations were enzymatically determined in neutralized extracts by fluorometric analysis (Lowry and Passonneau, 1972; Morales-Alamo et al., 2013).

### Electromyography

Electrical muscle activation was monitored using surface electromyography (EMG). EMG signals were continuously recorded from the *vastus medialis* and *vastus lateralis*. Before the application of the EMG electrodes the skin surface was carefully shaved and wiped with alcohol to reduce skin impedance. Bipolar single differential electrodes were placed longitudinally on the muscles following the SENIAM recommendations (Merletti and Hermens, 2000) and taped to the skin to minimize movement artifacts. The reference electrode was placed on the skin over the acromion. The position of the electrodes was marked on the skin with indelible ink, and these references were used for precise electrode placement on repeated experiments.

The EMG signals were acquired using a 16-channel recording system (Myomonitor IV, Delsys Inc., Boston, MA) at a sampling rate of 1000 Hz using rectangular shaped (19.8 mm wide and 35 mm long) bipolar surface electrodes with 1 × 10 mm 99.9% Ag conductors, and with an inter-conductor distance of 10 mm (DE-2.3 Delsys Inc.). The EMG data were filtered with a high-pass filter of 20 Hz and a low-pass filter of 450 Hz using a fifth-order Butterworth filter. The system has an input impedance of > 10^15^Ω per 0.2pF of input capacitance, a common mode rejection ratio of >80 dB, signal-to-noise ratio < 1.2 μV, and a pre-amplifier gain 1000 V/V ±1%. Each pedal revolution was detected using an electrogoniometer (Goniometer Biosignal Sensor S700 Joint Angle Shape Sensor; Delsys Inc. Boston) fixed on the left knee and sampled at 500 Hz. EMG and joint movement were simultaneously recorded by a portable device (Myomonitor IV, Delsys Inc. Boston) and wirelessly transmitted to a computer (EMGWorks Wireless application and EMGWorks Acquisition 3.7.1.3; Delsys, Inc. Boston).

The EMG signal corresponding to each muscle contraction was analyzed using code developed “in house” (Matlab R2012b, MathWorks, Natick, MA, USA). The EMG recordings were full-wave rectified and to provide an index of muscle activation, the amplitude characteristics were analyzed via average RMS of a 25-ms moving window for the duration of the burst. Burst onset and offset detection were determined using 20% of the maximal EMG_RMS_ activity of each burst as a reference (Baum and Li, 2003; Hug and Dorel, 2009; Torres-Peralta et al., 2014), rather than a mean threshold value from 15 consecutive bursts (Ozgünen et al., 2010). This approach yielded the same result as direct simple visual discrimination with 100% detection of all bursts. The EMG_RMS_ recorded during the last minute of a 2 min 80 W load (in normoxia) was used to normalize the remaining EMG_RMS_ data. Besides, we defined a total activity index during the sprint (TAI) as TAI = EMG_RMS_ × burst duration (ms) × number of pedal strokes during the sprint. The total activity index is similar to the integrated EMG signal, but was computed separately for each burst and excluded the baseline EMG between burst (Torres-Peralta et al., 2014). The TAI recorded during the last minute of a 2 min 80 W load (in normoxia) was used to normalize the rest of the TAI values.

The mean (MPF) and median (MdPF) power spectrum frequencies were calculated using the Fast Fourier Transform (Solomonow et al., 1990). All variables were reported as the mean values of the pedal strokes recorded during the last 10 s of the incremental exercise or the 10 s sprints. EMG variables responded similarly during the four control sprints. Therefore, to reduce EMG variability the four control sprints were averaged. EMG data are reported separately for *vastus medialis* and *lateralis*, and also as the average of the two muscles. Since the incremental exercise tests in normoxia and hypoxia were repeated, the mean of each pair was used in further analysis to represent either normoxia or hypoxia.

### Statistics

Normal distribution of variables was checked with the Shapiro-Wilks test. A repeated-measures ANOVA with F_I_O_2_ condition (normoxia vs. hypoxia) and occlusion duration (10 vs. 60 s) was used to analyze the responses observed during the sprints. Pairwise comparisons at specific time points were performed with Student t-tests, and adjusted for multiple comparisons using the Holm–Bonferroni method. Values are reported as the mean ± standard deviation (unless otherwise stated). *P* ≤ 0.05 was considered statistically significant. All statistical analyses were performed using SPSS v.15.0 for Windows (SPSS Inc., Chicago, IL) and Excel 2011 (Microsoft, Redmond, WA, USA).

## Results

### Incremental exercise

Compared to normoxia, Wmax, pulmonary ventilation, respiratory rate, heart rate and VO_2_peak were reduced during the last 10 s of exercise in hypoxia. Additional information regarding the respiratory and cardiovascular responses to the IE can be found elsewhere (Calbet et al., 2015; Morales-Alamo et al., 2015). The ergospirometric variables corresponding to the last 10 s of incremental exercise are reported in Table 1, together with the corresponding electromyographic responses.

**Table 1:** **Ergospirometric and electromyographic responses during the last 10 s of the incremental exercise to exhaustion in normoxia (P_I_O_2_ ≈ 143 mmHg) and severe hypoxia (P_I_O_2_ ≈ 73 mmHg)**.

	**Normoxia**	**Hypoxia**	***P***
F_I_O_2_ (%)	20.8±0.1	10.8±0.1	<0.001
SpO_2_(%)	93.1±4.4	64.6±4.6	<0.001
Wmax (W)	259.2±32.0	170.1±21.0	<0.001
VO_2_peak (l.min^−1^)	3.62±0.37	2.41±0.29	<0.001
V_E_ (l.min^−1^)	143.5±19.5	123.8±22.5	<0.001
RR (breaths.min^−1^)	60.2±7.6	53.8±7.0	<0.001
HR (beats.min^−1^)	185.4±6.0	175.1±9.0	<0.001
P_ET_O_2_(mmHg)	113.2±2.3	58.6±2.8	<0.001
P_ET_CO_2_(mmHg)	32.1±2.6	27.5±2.1	<0.001
RER	1.15±0.03	1.35±0.11	<0.001
VCO_2_(l.min^−1^)	4.12±0.49	3.23±0.41	<0.001
Pedaling rate (rpm)	56.7±7.4	55.4±5.9	0.5
VM RMSraw (μV)	134.2±60.1	113.3±48.9	<0.05
VL RMSraw (μV)	103.0±30.9	88.9±30.9	0.12
Average RMSraw (μV)	121.0±37.7	101.1±33.7	<0.01
VM RMSNz (A.U.)	219.1±77.6	182.3±45.5	<0.05
VL RMSNz (A.U.)	193.7±76.4	165.0±62.4	0.07
Average RMSNz (A.U.)	206.4±61.2	173.7±41.8	<0.05
VM TAINz (A.U.)	289.5±109.6	222.6±88.1	<0.001
VL TAINz (A.U.)	246.5±102.2	187.5±73.1	<0.05
Average TAINz (A.U.)	268.0±80.1	205.0±68.2	<0.001
VM MPF (Hz)	84.2±18.4	89.6±18.0	0.13
VL MPF (Hz)	84.1±18.6	89.6±17.8	0.13
Average MPF (Hz)	84.2±18.5	89.6±17.9	0.13
VM MdPF (Hz)	68.6±12.3	73.3±14.8	<0.05
VL MdPF (Hz)	68.5±12.5	73.0±14.6	<0.05
Average MdPF (Hz)	68.6±12.4	73.1±14.7	<0.05
VM Burst (ms)	401.9±51.7	362.5±55.9	0.12
VL Burst (ms)	401.4±55.2	368.3±55.7	0.10
Average Burst (ms)	401.7±49.4	365.4±52.8	0.10

*F_I_O_2_, inspiratory oxygen fraction; SpO_2_, hemoglobin saturation in capillary blood measured by pulse oximetry; Wmax, power output at exhaustion; VO_2_, oxygen consumption; V_E_, pulmonary ventilation; RR, respiratory rate; HR, heart rate; P_ET_O_2_, end-tidal O_2_ pressure; P_ET_CO_2_, end-tidal CO_2_ pressure; RER, respiratory exchange ratio; VCO_2_, CO_2_ production; rpm, revolutions per minute; VL, vastus lateralis; VM, vastus medialis; RMSraw, raw root mean square; RMSNz, normalized root mean square; TAINz, Normalized total activation index (arbitrary units, A.U.); MPF, mean power frequency; MdPF, median power frequency; Burst, burst duration; Average, mean of VM and VL*.

### Muscle fatigue

**Figure 3:**
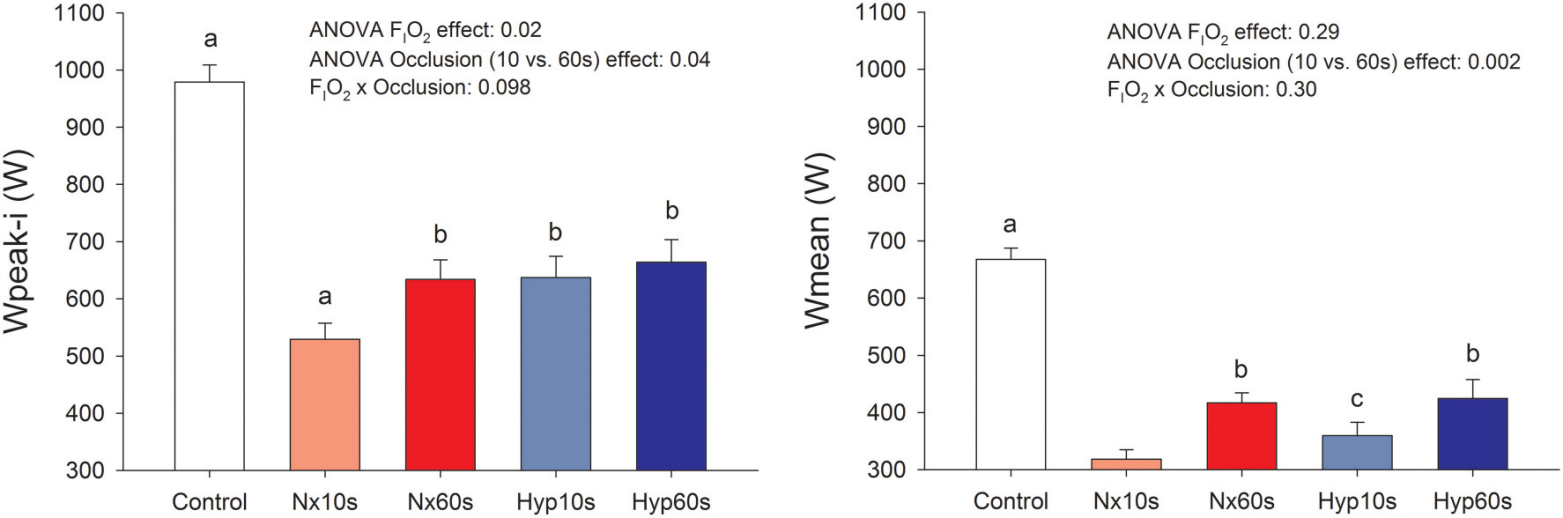
**Power output**. Peak (Wpeak-i) and mean (Wmean) power output during sprint exercise performed at the end of an incremental exercise to exhaustion in normoxia (P_I_O_2_ ≈ 143 mmHg) and severe hypoxia (P_I_O_2_ ≈ 73 mmHg), after 10 or 60 s of occlusion of the circulation. ^a^*P* < 0.05 compared with the other conditions; ^b^*P* < 0.05 compared with Nx10s; ^c^*P* < 0.05 compared with Nx60s.

Compared to the control sprints, post-IE sprint performance was reduced (32–46%, *P* < 0.05; Figure 3) as previously reported (Morales-Alamo et al., 2015). Sprint Wpeak-i was 11% lower following post-IE in normoxia than hypoxia. Similar effects were observed in Wmean. Sprint performance was improved after ischemic recovery. Wpeak-i and Wmean were 11 and 23% higher, respectively, following 60 than 10 s of occlusion (*P* < 0.05), as previously reported (Morales-Alamo et al., 2015).

### EMG responses

#### *Vastus lateralis* EMGs

Figure 2 depicts an example of the EMG recordings during one experimental day. During the last 10 s of the incremental exercise to exhaustion the raw (average of the two muscles) and normalized RMS were 16% lower in hypoxia than in normoxia (*P* < 0.05) (Table 1). The average normalized TAI was also lower (23%) in hypoxia than in normoxia (*P* < 0.001). The median frequency was 6% lower in normoxia than hypoxia (*P* < 0.05). The mean and median frequencies during the last 10 s of the IE remained at the same level during the subsequent sprints.

The average RMS, RMSNz, and TAINz were 1.6-, 1.7-, and 2.9-fold higher during the sprints post-IE than during the last 10 s of the IE (all, *P* < 0.05). Compared to the control sprints, the RMSraw and the normalized RMSNz were reduced by 36 and 35%, respectively, in the sprints performed after the IE (Table 2). Although the 60 s occlusion caused a 14% lower RMSNz compared to the 10 s occlusion, this difference did not reach statistical significance (Main effect *P* = 0.059). Compared to the control sprints the TAINz was reduced by 42 and 34% during the sprints performed after the incremental test in normoxia and hypoxia, respectively (*P* < 0.05).

**Table 2:** ***Vastus lateralis* electromyographic variables in response to sprint exercise performed at the end of an incremental exercise to exhaustion in normoxia (P_I_O_2_ ≈ 143 mmHg) and severe hypoxia (P_I_O_2_ ≈ 73 mmHg), after 10 or 60 s of occlusion of the circulation**.

	**Control sprint**	**Nx10s**	**Nx60s**	**Hyp10s**	**Hyp60s**	**Main Oxy (*P*)**	**Main Occ (*P*)**	**Interaction Oxy × Occ (*P*)**
RMSraw (μV)	238.1±82.0^a^	153.2±64.7	137.1±53.2	160.2±68.7	161.8±61.8	0.220	0.706	0.429
RMSNz (A.U.)	475.1±155.2^a^	317.3±95.1	254.6±110.0	344.2±126.4^c^	317.0±172.4	0.112	0.059	0.436
Burst (ms)	331.9±24.6^b^^,^^d^	299.7±22.7^c^	324.6±27.7	305.5±23.8^c^	337.3±34.4^b^^,^^d^	0.026	0.001	0.381
TAINz (A.U.)	1023.1±340.6^a^	600.8±268.8	581.5±244.2	642.9±297.0	715.8±288.2	0.12	0.729	0.326
MPF (Hz)	93.9±9.7^b^^,^^c^	81.9±14.2	79.5±16.4	86.3±20.3	89.5±17.2	0.006	0.837	0.212
MdPF (Hz)	80.2±6.0^b^^,^^c^^,^^d^	66.4±9.4^e^	67.0±13.6	71.4±16.6	74.7±13.9^c^	0.011	0.255	0.500

a *P < 0.05 compared with the other conditions*.

b *P < 0.05 compared with Nx10s*.

c *P < 0.05 compared with Nx60s*.

d *P < 0.05 compared with Hyp10s*.

e *P < 0.05 compared with Hyp60s*.

*RM ANOVA (2 × 2) Main Oxy: main oxygenation effect due to the conditions in which was performed the incremental exercise test (Nx, Normoxia; Hyp, hypoxia); RM ANOVA (2 × 2) Main Occ, Main Occlusion effect due to the duration of the occlusion (10 vs. 60 s); Wpeak-i, instantaneous peak power in the sprint; Wmean, mean power in the 10 s sprint; RMSraw, raw root mean square; RMSNz, Normalized root mean square; Burst, burst duration; TAINz, Normalized total activation index (arbitrary units, A.U.); MPF, mean power frequency; MdPF, median power frequency*.

Compared to the control sprint, the median frequency was reduced by 18 and 9% in the sprints that followed the IEs in normoxia and hypoxia, respectively (both, *P* < 0.05) (Table 3). Thus, the median frequency was 9% lower in the sprints that followed IE in normoxia than in hypoxia (Main effect *P* = 0.011). MPF changes were essentially similar to MdPF (Tables 1–4).

**Table 3:** ***Vastus medialis* electromyographic variables in response to sprint exercise performed at the end of an incremental exercise to exhaustion in normoxia and severe hypoxia (P_I_O_2_ ≈ 73 mmHg), after 10 or 60 s of occlusion of the circulation**.

	**Control sprint**	**Nx10s**	**Nx60s**	**Hyp10s**	**Hyp60s**	**Main Oxy (*P*)**	**Main Occ (*P*)**	**Interaction Oxy × Occ (*P*)**
RMSraw (μV)	263.5±115.7^b^^,^^c^^,^^e^	177.4±95.0^d^	193.9±121.3	204.2±103.1	183.9±81.1	0.333	0.943	0.038
RMSNz (A.U.)	410.4±163.3^a^	284.0±97.1^d^	276.4±126.1	333.0±143.1	279.7±104.8^d^	0.078	0.137	0.098
Burst (ms)	342.2±27.7^b^	309.4±42.5^c^	355.2±49.4^d^	322.0±53.1	347.8±37.9^b^	0.743	0.017	0.154
TAINz (A.U.)	1152.1±463.3^a^	701.1±359.9^d^	809.0±498.6	829.7±397.9	820.7±335.6	0.118	0.638	0.119
MPF (Hz)	94.3±9.9^b^^,^^c^^,^^d^	81.9±14.0	79.8±15.8	85.8±19.2	89.4±17.4	0.008	0.703	0.241
MdPF (Hz)	80.8±6.7^a^	66.2±9.2	67.1±13.4	71.3±16.6	74.6±14.1^b^^,^^c^	0.011	0.218	0.587

a *P < 0.05 compared with the other conditions*.

b *P < 0.05 compared with Nx10s*.

c *P < 0.05 compared with Nx60s*.

d *P < 0.05 compared with Hyp10s*.

e *P < 0.05 compared with Hyp60s*.

*RM ANOVA (2 × 2) Main Oxy, main oxygenation effect due to the conditions in which was performed the incremental exercise test (Nx, Normoxia; Hyp, hypoxia); RM ANOVA (2 × 2) Main Occ, Main Occlusion effect due to the duration of the occlusion (10 vs. 60 s); RMSraw, raw root mean square; RMSNz, Normalized root mean square; Burst, burst duration; TAINz, Normalized total activation index (arbitrary units, A.U.); MPF, mean power frequency; MdPF, median power frequency*.

**Table 4:** **Electromyographic variables (average of *vastus medialis* and *lateralis*) in response to sprint exercise performed at the end of an incremental exercise to exhaustion in normoxia (P_I_O_2_ ≈ 143 mmHg) and severe hypoxia (P_I_O_2_ ≈ 73 mmHg), after 10 or 60 s of occlusion of the circulation**.

	**Control**	**Nx10s**	**Nx60s**	**Hyp10s**	**Hyp60s**	**Main Oxy (*P*)**	**Main Occ (*P*)**	**Interaction Oxy × Occ (*P*)**
RMSraw (μV)	250.8±89.1^a^	165.3±69.6^d^	165.5±76.5	182.2±79.8	172.9±50.7	0.164	0.793	0.536
RMSNz (A.U.)	442.7±141.9^a^	300.6±88.9^d^	265.5±93.9^d^	338.6±122.7	298.3±117.7^d^	0.042	0.043	0.835
Burst (ms)	337.1±23.9^b^^,^^d^	304.5±29.8^c^	339.9±36.0^d^	313.7±36.0	342.6±35.3^b^^,^^d^	0.305	0.005	0.754
TAINz (A.U.)	1089.7±355.0	653.1±274.8^d^	720.1±323.5	739.6±331.4	769.7±238.9	0.082	0.510	0.575
MPF (Hz)	94.1±9.8^b^^,^^c^^,^^d^	81.9±14.1	79.6±16.1	86.0±19.7	89.5±17.3^b^^,^^c^	0.007	0.769	0.221
MdPF (Hz)	80.5±6.3^b^^,^^c^^,^^d^	66.3±9.2	67.0±13.5	71.3±16.6	74.6±14.0^b^^,^^c^	0.011	0.234	0.543

a *P < 0.05 compared with the other conditions*.

b *P < 0.05 compared with Nx10s*.

c *P < 0.05 compared with Nx60s*.

d *P < 0.05 compared with Hyp10s*.

*RM ANOVA (2 × 2) Main Oxy, main oxygenation effect due to the conditions in which was performed the incremental exercise test (Nx, Normoxia; Hyp, hypoxia); RM ANOVA (2 × 2) Main Occ, Main Occlusion effect due to the duration of the occlusion (10 vs. 60 s); RMSraw, raw root mean square; RMSNz, Normalized root mean square; Burst, burst duration; TAINz, Normalized total activation index (arbitrary units, A.U.); MPF, mean power frequency; MdPF, median power frequency*.

Compared to the control sprints, the duration of the bursts was reduced by 9% in the sprints performed after 10 s occlusions. Nevertheless, after 60 s of occlusion, the duration of burst in the following sprints achieved the same value as in the control sprints (331.9 ± 24.6 and 331.0 ± 29.7 ms, control and 60 s after IE, respectively, *P* = 0.91). The duration of the bursts was 3% longer in the sprints that followed an IE in hypoxia than normoxia (*P* = 0.03). The average contraction time (i.e., burst duration x pedaling rate) was 15% lower during the last 10 s of IE than during the post-IE sprints (*P* < 0.001).

#### *Vastus medialis* EMGs

The *vastus medialis* EMG responses to incremental (Table 1) and sprint exercise (Table 3) were similar to those described for the *vastus lateralis*. Therefore, we combined both *vastus* EMGs responses to reduce variability (Table 4). The combined response was similar to that reported for *vastus lateralis* and *medialis*, but with lower variability, confirming some of the effects that did not reach statistical significance when only analyzed using a single muscle EMG recordings.

### Muscle metabolites

Muscle metabolites and the aerobic and anaerobic energy yield during the sprints have been reported elsewhere (Morales-Alamo et al., 2015). Briefly, ATP and PCr concentrations were reduced to a similar extent both in normoxia and hypoxia (ANOVA time effect: *P* < 0.05). From the 10th to 60th s of ischemic recovery ATP concentration remained unchanged while PCr declined an additional ~5% (ANOVA time effect: *P* < 0.05 compared to Post and *P* < 0.001, compared to PRE). Muscle lactate concentration was increased to similar values 10 s after both incremental exercise tests (93.5 ± 24.3 and 88.3 ± 26.6 mmol kg d.w.^−1^, in normoxia and hypoxia, respectively (ANOVA time effect: *P* < 0.001). From the 10th to 60th s of ischemia muscle lactate was increased by 24.0 ± 20.7 and 21.6 ± 24.5 mmol kg d.w.^−1^, and muscle pH reduced by 0.102 ± 0.040 and 0.109 ± 0.041 units, after the IE in normoxia and hypoxia, respectively (ANOVA time effect: *P* < 0.01).

## Discussion

This investigation confirms, in agreement with previous studies using constant intensity (Marcora and Staiano, 2010) and incremental exercise to exhaustion in normoxia (Coelho et al., 2015), that task failure during incremental exercise in normoxia is not caused by muscle fatigue. The present study extends these findings to whole-body incremental exercise in severe hypoxia. We have also shown that muscle activation during the last 10 s of an incremental exercise to exhaustion is lower in severe hypoxia than in normoxia. This reduction in muscle activation cannot be explained by differences in metabolite accumulation between hypoxia and normoxia, and likely reflects central mechanisms of fatigue, which recovered, at least partly, during the next 10 or 60 s of ischemia, as reflected by the greater activation of the muscles during the subsequent sprint. The latter occurred despite the lower energy availability and the greater accumulation of metabolites after the ischemic recoveries. We have shown that neuromuscular performance, as assessed by a sprint test, at the end of an incremental exercise to exhaustion, is greater when the incremental exercise has been performed in severe acute hypoxia than in normoxia despite similar muscle metabolite accumulation. This is also compatible with the incremental exercise in hypoxia ending by central mechanism/s acting earlier, i.e., with a lower amount of peripheral fatigue in hypoxia than in normoxia. This reduction in sprint performance is accompanied by a greater reduction in muscle activation (as reflected by the EMG_RMS_ changes) in the sprints carried out after IE in normoxia than hypoxia. This could also be interpreted as indicative of slower recovery of central mechanisms of fatigue during the 10 s of ischemic recovery that followed the IE performed in normoxia compared to the sprint performed after the IE in hypoxia.

Strikingly, following 60 s of recovery with complete occlusion of the circulation sprint performance was higher than that observed immediately following 10 s of occlusion, as previously reported (Morales-Alamo et al., 2015). This improvement in performance was achieved with lower EMG_RMS_ compared to the sprints executed after 10 s of ischemia. Thus, this investigation demonstrates dissociation between the recovery of the EMG_RMS_ and the recovery of power output. Since occlusion resulted in an increase of muscle lactate and H^+^ accumulation from 10 to 60 s of occlusion (Morales-Alamo et al., 2015), the present experiments support the concept that muscle acidification contributes to reduce EMG_RMS_. In addition, we have demonstrated that despite similar muscle concentrations of lactate and H^+^ at the beginning of the respective sprints, the MPF and MdPF during sprint exercise are reduced by muscle fatigue when the sprint is performed after an incremental exercise in normoxia, but not in hypoxia. MPF and MdPF did not recover from the end of the incremental exercise to the start of the sprints, or from the 10th to the 60th second of ischemia, despite cessation of neural activation and appropriate oxygenation of the brain. These results point to peripheral mechanisms being primarily responsible for the reduction of MPF and MdPF during the sprints post-IE (Juel, 1988; Brody et al., 1991). Moreover, despite greater lactate and H^+^ accumulation after 60 s of ischemic recovery, sprint exercise MPF and MdPF were not further reduced; therefore these two metabolites do not seem to account for the observed reduction of MPF and MdPF with muscle fatigue (Vestergaard-Poulsen et al., 1995). We have also shown that the duration of the bursts is reduced in fatigued muscles contracting under isokinetic conditions, recovering to non-fatigued levels within 60 s, regardless of the muscular concentrations of lactate and H^+^. We lastly provided evidence for a minor impact, if any, of increased group III/IV afferent feedback on sprint exercise performance.

### Surface EMG changes are more sensitive to central than peripheral fatigue

To demonstrate the existence of central fatigue it is necessary to show that during a maximal voluntary contraction, direct stimulation of motor neural pathways or cortical motoneurons results in greater levels of force than elicited voluntarily (Merton, 1954; Gandevia et al., 1996). These procedures have several constraints limiting their application to complex tasks, such as pedaling. To circumvent these problems, a commonly used approach has been to carry out potentiated and interpolated twitch assessments at exhaustion as fast as possible, i.e., 1–2 min after the task failure, when most of the reduction in power output has already been recovered (Sargeant and Dolan, 1987; Fernandez-del-Olmo et al., 2013; Froyd et al., 2013; Coelho et al., 2015). Another limitation of stimulation techniques is that muscle fatigue is task-specific (Gandevia, 2001). Stimulation techniques cannot reproduce the complexity of motor orders involving thousands of motor units from different muscles firing at different rates, which are recruited with specific timings to achieve a coordinated movement. Also, in fatigued muscles, the increase in force elicited by an interpolated twitch may be due in part to intracellular mechanisms (i.e., a peripheral mechanism that is likely related to the force/[Ca^2+^]_i_ relationship) (Gandevia et al., 2013), not necessarily reflecting increased central fatigue.

An alternative approach for assessing muscle fatigue is to measure the maximal power that can be generated during the task that elicits muscle fatigue (Cairns et al., 2005; Marcora and Staiano, 2010; Coelho et al., 2015). However, during whole-body exercise on the cycle ergometer, pedaling frequency slows down close to task failure (Torres-Peralta et al., 2014). Given the dependency of power on muscle contraction velocity, fatigue should ideally be tested under similar muscle contraction velocities as during isokinetic pedaling (Sargeant and Dolan, 1987).

In the present experiments, the sprints were performed in isokinetic conditions, i.e., the duration of each pedaling cycle was always the same, regardless of the state of muscle fatigue (Figure 2). This isokinetic approach was possible because the cycle ergometer servo-control instantaneously varied the resistance applied to the pedals depending on the force exerted resulting in a constant pedaling rate of 80 rpm. These conditions allowed us to examine the impact of fatigue on certain components of the EMG signal without the variability induced by the speed of movement.

At a given pedaling rate, the duration of contraction bursts increases with the intensity of exercise, reaching maximal values at intensities close to Wmax (Torres-Peralta et al., 2014). In all conditions and every subject, the mean intensity achieved during the 10 s sprints was above the intensity reached at exhaustion during the IEs. This result implies that concerning intensity, burst duration should have been maximal during all sprints. However, burst duration during the sprints performed after 10 s of ischemic recovery was reduced, meaning that muscles were activated during a shorter fraction of the pedaling cycle compared to the control sprints. This effect might have been caused by reduced sarcolemmal excitability as a consequence of fatigue (Sejersted and Sjogaard, 2000; Sidhu et al., 2012). Reduced sarcolemmal excitability during hand grip exercise has been associated with lactic acidosis in blood (Hilbert et al., 2012). However, if present at exhaustion, sarcolemmal excitability appears to recover in less than 60 s after a fatiguing sprint exercise (Fernandez-del-Olmo et al., 2013). In agreement, burst duration recovered within the 60 s of ischemic rest after both IEs. Despite the observed recovery of burst duration, the EMG_RMS_ was further reduced following 60 s of ischemia. Since the reduction in EMG_RMS_ was accompanied by increased power output and normal burst duration, it seems unlikely that the lower sprint EMG_RMS_ after 60 s of ischemia originates from a failure of the muscle cells to respond to neural activation.

During high-intensity contractions, the myosin ATPase and ion pumps account for most of the energy expenditure in muscles because no energy can be diverted to biosynthetic processes as the required enzymes are blocked (Morales-Alamo and Calbet, 2014). In our experimental conditions, upon cessation of incremental exercise the ion pumps are expected to be maximally activated, particularly the Na^+^-K^+^ pump, which has a critical role in restoring sarcolemmal excitability (Pedersen et al., 2003; Hostrup et al., 2014). During ischemia, the energy needed to maintain the activity of the ion pumps was provided by the glycolysis and to a lesser extent by the minute amount of remaining PCr (Morales-Alamo et al., 2015). The O_2_ that still remained bound to myoglobin at the end of incremental exercise was rapidly used upon occlusion due to the strong activation of mitochondrial respiration by the increased ADP concentration at task failure (Morales-Alamo et al., 2015). Femoral vein and mean capillary PO_2_ were lower at exhaustion after the incremental exercise test in severe hypoxia than in normoxia (Calbet et al., 2015). Therefore, the potential contribution of the small amount of O_2_ trapped in the occluded leg (or remaining bound to myoglobin) to ATP re-synthesis should have been lower in hypoxia than in normoxia. However, although more O_2_ was available at exhaustion for the initial recovery in normoxia, i.e., within the first 10 s of occlusion, performance was more impaired in the sprint performed after 10 s of ischemic recovery that followed the normoxic rather than the hypoxic IEs. This result concurs with task failure occurring with a lower level of peripheral fatigue during IE in hypoxia than normoxia, as our results indicate.

Studies in cats (Hill et al., 1992; Lagier-Tessonnier et al., 1993) and rabbits (Arbogast et al., 2000) have shown increased baseline discharge frequency of group III and IV muscle afferents by PO_2_ close to the values observed in the femoral vein in the present investigation (Calbet et al., 2015). Thus, lower interstitial PO_2_ values in hypoxia than normoxia could have enhanced III/IV afferent feedback to cause increased inhibition of the corticospinal drive during exercise in severe acute hypoxia, as previously suggested (Calbet et al., 2015). Muscle III and IV afferent, and perhaps other sensory endings in joints and tendons, have been postulated to contribute to central fatigue (Reid, 1927; Bigland-Ritchie et al., 1986; Garland, 1991; Amann et al., 2008, 2013a). Animal studies have shown that when motoneurons are stimulated repetitively many neurons reduce their discharge frequency or stop firing (Kernell and Monster, 1982a,b; Spielmann et al., 1993). This phenomenon is likely accentuated by hypoxia. Consequently, reduced responsiveness of some motoneurons pools (central fatigue) combined with increased III/IV muscle afferent feedback and increased ventilatory demand at any given absolute intensity might have increased the perception of effort (Marcora, 2009), leading to task failure at the end of IE in hypoxia with a lower level of peripheral fatigue than in normoxia (Pierrefiche et al., 1997). Oxygenation upon exhaustion might have restored faster central fatigue at the end of the IE in hypoxia than normoxia, by reinstating within seconds normoxic interstitial PO_2_ levels in the CNS. In agreement, with altitude acclimatization, oxygen delivery at VO_2_max is almost restored to sea level values (Calbet and Lundby, 2009), improving brain oxygenation compared to acute hypoxia and reducing supraspinal fatigue (Goodall et al., 2014a,b).

The absolute exercise intensity at task failure was lower in hypoxia than normoxia. However, metabolite accumulation was similar regardless of F_I_O_2_ and, hence, differences in absolute exercise intensity do not seem to account for the differences in peripheral fatigue.

### Group III and IV muscle afferent stimulation does not limit peak power output during whole-body sprint exercise in healthy humans

Group III/IV muscle afferent neurons include a complex family of afferent endings some of which act as nociceptors while others respond to thermal, mechanical or chemical stimulation (Light et al., 2008; McCord and Kaufman, 2010; Jankowski et al., 2013). Metabolic products of muscle contraction like H^+^ (Rotto and Kaufman, 1988; Light et al., 2008), lactate (Light et al., 2008), adenosine (Middlekauff et al., 1997), ATP (Light et al., 2008), nitric oxide (Arbogast et al., 2001), and inflammation mediators may also increase III/IV muscle afferent discharge (Light et al., 2008; McCord and Kaufman, 2010). It is particularly important that the response to the isolated increase of H^+^, ATP and lactate is much lower than observed to the combination of the three (Light et al., 2008). Some metaboreceptive III and IV muscle afferents seem specialized in detecting innocuous levels of metabolites, while others respond to noxious levels and contribute to muscle pain (Kniffki et al., 1978; Mense, 1996). It is thought that both low and high concentration responding-endings could contribute to sympathetic reflexes and to increase the perception of effort (Light et al., 2008).

Using lumbar intrathecal fentanyl administration before exercise to block μ-opioid receptor-sensitive group, Amann and co-workers (Amann et al., 2009, 2011) studied the influence of group III and IV afferents on voluntary activation (assessed by the twitch-interpolation technique) after constant-intensity bicycling exercise. After 3 min of recovery, voluntary activation was reduced by 1.2% during the placebo trial (non-significant) and 1.7% during the fentanyl trial (Amann et al., 2011). Moreover, 3 min after a time trial that caused fatigue in 7.5 min, voluntary activation was reduced by 1.6% (compared to 0.8% in the placebo trial) (Amann et al., 2009). These results further emphasize that any potentially negative influence of III and IV muscle afferents on voluntary activation is likely small after whole-body exercise or that voluntary activation capacity recovers rather quickly (Bigland-Ritchie et al., 1986; Kennedy et al., 2015; Pageaux et al., 2015).

Amann et al. (2011) reported that despite the inhibition of III and IV muscle afferents, fentanyl markedly reduced performance (from 8.7 to 6.8 min) during a constant-intensity trial to exhaustion. In another study by the same group (Amann et al., 2009), where the subjects performed a time trial, performance was similar in the fentanyl and placebo experiments. The reason for these discrepant effects of III/IV muscle afferents inhibition on performance is not clear (Marcora, 2010). In the case of the constant-intensity trial, there was a substantial impairment in O_2_ transport in the fentanyl trial, an effect caused by reduced ventilation, leading to lower hemoglobin saturation and VO_2_ with fentanyl than placebo. A slightly lower impairment of O_2_ transport was reported in the time trial experiments, which could have been compensated for by increased involvement of the anaerobic metabolism (Amann et al., 2009). Marcora (2010) have suggested that during the time trial, the “belief effect” induced by reducing leg muscle pain with fentanyl-induced a fast start, which compensated for the negative effect of fentanyl on cardiorespiratory responses. As a result, exercise performance was unchanged during the time trial. During the constant-intensity trial, the “belief effect” could not induce a fast start and consequently exercise performance was reduced.

Interestingly, in both experiments with fentanyl the reduction of quadriceps potentiated twitch forces elicited by magnetic neural stimulation were greater than in the control trials (Amann et al., 2009, 2011). Assuming that a reduction in the force elicited by potentiated twitches indicates a greater level of peripheral fatigue and that the subjects exercised to their limits in all trials, Amann and co-workers studies give support to the concept that III/IV afferent feedback is used to set the limit of peripheral fatigue that is permitted. However, these experiments, combined with the present findings, show that task failure at the end of an IE to exhaustion (current study), or at the end of constant-intensity (Marcora and Staiano, 2010; Bosio et al., 2012) or a simulated time-trial competition (Amann and co-workers studies) is not due to a peripheral failure. Both types of experiments show that greater levels of peripheral fatigue are possible, but not reached because the exercise ends due to central mechanisms of fatigue (Kayser, 2003). The critical question is whether peripheral fatigue causes central fatigue and task failure through a central mechanism by activating the metaboreflex. If the latter is the main mechanism causing central fatigue and task failure a reduction in sprint performance under conditions with increased metaboreflex activation will be expected. However, our results show that this is not the case.

Metabolite accumulation at task failure and during ischemia should have promoted a sustained discharge of III/IV muscle afferents (Darques and Jammes, 1997; Darques et al., 1998; Light et al., 2008; McCord and Kaufman, 2010), an effect that would be expected to be greater after 60 than 10 s occlusions in the present investigation. Nevertheless, sprint exercise power output was higher after 60 than 10 s of ischemia. Increased metaboreflex activation should exacerbate the ventilatory response to exercise (Amann et al., 2010), as observed during the sprints performed following 60 s of ischemia in the present investigation (Morales-Alamo et al., 2015). Thus, despite increased metaboreflex activation during the sprints after 60 s of ischemia, and a theoretically worsened metabolic situation, peak and mean power output was higher during the sprints after 60 than 10 s of ischemia. Our findings appear to be at odds with the recent study by Kennedy et al. (2015), in which the occlusion of the circulation during 2 min after a 2-min sustained MVC of the knee extensors resulted in a progressive reduction of the maximal voluntary contraction (MVC) (see Figure 3A of Kennedy et al., 2015). This effect was accompanied by a progressive reduction of maximal voluntary activation indicative of increasing central fatigue (see Figure 3B of Kennedy et al., 2015). In agreement with our results, Kennedy et al. (2015) observed a fast recovery of voluntary activation upon release of the cuff, indicating that the negative influence of III/IV afferent discharge on voluntary activation was also relieved very rapidly. This quick response could be due to (i) the release of the direct compression on the femoral nerve, (ii) the washout of metabolites combined with oxygenation due to reactive hyperemia, or (iii) the combination of these effects. In our experimental conditions, the hyperemic response to the release of the cuff was likely much greater than in Kennedy et al. (2015) as reflected by the rapid increase of pulmonary VO_2_ and the elevated heart rate at the beginning of the sprint (Morales-Alamo et al., 2015). Another interesting aspect of the study by Kennedy et al. (2015) is that the level of peripheral fatigue as assessed by potentiated twitches remained at the same level during the 2 min of ischemia, despite 5 × 2-s long MVC maneuvers (i.e., 10 s of maximal contractile activity with occlusion of the circulation). Thus, the 2 min of post-exercise ischemia did not worsen peripheral fatigue (Kennedy et al., 2015). Although Kennedy et al. (2015) did not measure muscle metabolites, 10 s of maximal contractile activity during the 2 min of ischemia, in combination with increased metabolic demand due to the preceding MVC (sustained during 2 min), should have increased peripheral fatigue according to the prevailing paradigm.

The present investigation strongly suggests that the inhibitory role of muscle afferent discharge on the corticospinal motor drive during maximal intensity whole-body exercise is either small or counteracted by a strong corticospinal drive (central command). Otherwise, the III/IV muscle afferent feedback due to ischemia (PO_2_ close to 0 mmHg in our experimental conditions) and the metabolites accumulated during the IEs should have decreased Wpeak and Wmean during the 10 or 60 s post-ischemia sprints below the Wmax achieved at task failure in the IEs. This finding contrasts with experiments using single joint (Gandevia et al., 1996; Kennedy et al., 2015) or handgrip dynamic contractions (Broxterman et al., 2015), where a clear inhibition of voluntary activation is consistently shown.

In the present investigation, increased III/IV muscle afferent feedback could explain why the observed EMG_RMS_ were lower in the sprints performed after 60 s than after 10 s of ischemia. Likewise, that MPF and MdPF remained at task failure levels during the post-IE sprints is also consistent with an on-going negative feedback reducing motoneurons firing frequencies (Broxterman et al., 2015). However, the power output achieved during the sprints after 60 s of ischemia is close to the maximal power attainable by our subjects without phosphagens (Morales-Alamo et al., 2015), leaving little room for central mechanisms to limit sprint performance after 60 s of ischemia. Thus, it seems that 60 s of ischemic recovery allows for restoration of the central mechanisms of fatigue, despite the discharge from the III/IV muscle afferents. The latter was likely counteracted by a strong central command and a rapid reperfusion of the muscle upon the release of the cuff during the post-IE sprints. In agreement with this interpretation, Amann et al. (2009) reported no effect of intrathecal fentanyl on time trial performance, although the power output profile was dramatically changed.

### Reduced sprint EMG_RMS_ but increased power output after 60 than 10 s post-exercise ischemia

*In vitro* experiments have shown that lactate and H^+^ accumulation may facilitate peripheral recovery by increasing chloride conductance (Nielsen et al., 2001; Pedersen et al., 2003; Karelis et al., 2004). Thus, an elevated glycolytic rate combined with a progressive rise of muscle temperature due to flow arrest during ischemic recovery could have exerted two opposing actions. Positively, increased glycolysis may have facilitated peripheral recovery by enhancing sarcolemmal excitability (Pedersen et al., 2003), which was likely depressed immediately after the IEs. Negatively, increased glycolysis and the subsequent metabolite accumulation may have increased III/IV muscle afferent discharge, interfering with muscle activation. The combination of both mechanisms could explain the lower EMG_RMS_ values observed during the sprints performed 60 s compared to 10 s after the IEs. If we assume that the normalized EMG_RMS_ is a valid index of corticospinal motor output, only reduced when the corticospinal motor drive is lower, a greater reduction in sprint EMG_RMS_ after prolonged (60 s) compared to shorter (10 s) ischemia would lead to the untenable conclusion that all of the improvement in Wpeak and Wmean observed from 10 to 60 s of ischemic recovery is due to peripheral mechanisms. Moreover, this peripheral-based improvement would have to be sufficient to counteract the reduced motor command. A more plausible explanation is that for a given level of neural activation, EMG_RMS_ is reduced by metabolite accumulation; i.e., EMG_RMS_ does not faithfully reflect the level of neural activation (Vestergaard-Poulsen et al., 1995). In agreement with this interpretation, it has been shown that central fatigue recovers rapidly upon cessation of contractile activity, even when peripheral fatigue remains at the same level (Bigland-Ritchie et al., 1986; Gandevia et al., 1996). Therefore, the observed reduction in sprint EMG_RMS_ after 60 s compared to 10 s of ischemic recovery is unlikely to be caused only by increased central mechanisms of fatigue.

### Fatigue reduces the duration of the burst during isokinetic sprints

The duration of the burst was similarly decreased in the sprints performed 10 s after the incremental exercise in hypoxia and normoxia. However, sprint power output was greater after the incremental exercise in hypoxia. To generate greater power, more mechanical impulse must be produced. As the duration of the bursts (a surrogate of contraction time) was similar in the sprints performed 10 s after both IEs, the subjects must have applied a greater force on the pedals after the hypoxic IE. Therefore, greater motor cortical drive was required in the sprint that occurred after hypoxia. In agreement, with this conclusion, the MPF and MdPF were higher in the sprints preceded by IE in hypoxia than normoxia, suggesting greater firing rates (Solomonow et al., 1990; Gerdle and Fugl-Meyer, 1992; Sbriccoli et al., 2003) and a lower degree of central fatigue during the sprint 10 s after the IE in hypoxia. Alternatively, central fatigue could have recovered more rapidly after the IE in hypoxia, likely due to a direct effect of oxygenation on the CNS, as previously postulated (Kayser et al., 1994; Calbet et al., 2003a,b; Amann et al., 2007; Goodall et al., 2014a).

### Limitations

At the end of the incremental exercise muscle activation was much lower than during the subsequent sprints, despite the fact muscles remained ischemic and that metabolite accumulation reduces EMG_RMS_. This finding indicates that task failure was not due to muscle fatigue; it was caused by reduced muscle activation. However, this conclusion is not based on genuine assessments of central fatigue and peripheral fatigue with stimulation techniques. Nonetheless, there is not an ideal method to quantify the extent of change in neuromuscular function (Cairns et al., 2005). Current methods based on stimulation techniques have also limitations. For example, the response of fatigued muscles to potentiated twitches depends on the method of stimulation (Froyd et al., 2013). Moreover, methods based on electrical or magnetic stimulation may give results different from those obtained using voluntary dynamic contractions. Few minutes after a 30 s all out sprint exercise (Wingate test) potentiated quadriceps twitches indicate substantial peripheral fatigue, when at the same time peak power output has completely recovered (Fernandez-del-Olmo et al., 2013).

In summary, task failure at the end of an incremental exercise to exhaustion depends more on central than peripheral mechanisms, as indicated by the fact that the level of muscle activation observed during the last 10 s of the incremental exercise was much lower than that reached 10 s later during a 10 s sprint, despite the fact that muscle remained ischemic from the end of exercise to the start of the sprint. The central components of fatigue appear to recover to a greater extent, during the first 10 s following an incremental exercise to exhaustion in hypoxia than in normoxia, concomitant with a rapid change to normoxic breathing during the recovery. We have also shown that ischemic recovery after incremental exercise to exhaustion allows for a partial restoration of power output despite increased acidification and reduced EMG_RMS_. Moreover, this study demonstrates that MPF, MdPF and the duration of the bursts during isokinetic sprints are reduced with fatigue. Lastly, this investigation indicates that increased group III/IV muscle afferent discharge has a minor, if any, negative impact on sprint exercise performance in healthy humans.

## Author contributions

Conception and design of the experiments: JC; pre-testing, experimental preparation, data collection and analysis: RT, DM, JL, IP, and JC; EMG analysis: RT, MG, and MI. The first version of the manuscript was written by RT and JC. All co-authors read, contributed comments and approved the final version of the manuscript.

## Conflict of interest statement

The authors declare that the research was conducted in the absence of any commercial or financial relationships that could be construed as a potential conflict of interest.

## Abbreviations

ADP: Adenosine diphosphate
ATP: Adenosine triphosphate
CNS: Central nervous system
d.w.: Dry weight
DEXA: Dual-energy x-ray absorptiometry
EMG: Surface electromyogram
EMG_RMS_: Root mean square of the EMG
F_I_O_2_: Inspired oxygen fraction
HR: Heart rate
HRmax: Maximal heart rate
Hyp: Hypoxia
Hypb: session in hypoxia with biopsies taken
IE: Incremental exercise to exhaustion
IS: Isokinetic sprint
MPF: Mean power frequency
MdPF: Median power frequency
MVC: Maximal voluntary contraction
Nx: Normoxia
Nxb: Session in normoxia with biopsies taken
PaO_2_: Arterial oxygen pressure
PCr: Phosphocreatine
P_I_O_2_: Inspired O_2_ pressure
RMS: root mean square
RMSNz: Normalized root mean square
V_E_: Minute ventilation
VO_2_: Oxygen consumption
VO_2_max: Maximal oxygen uptake
VO_2_peak: Peak oxygen uptake
Wpeak-i: Instantaneous peak power output
Wmax: Peak power output at exhaustion during the incremental exercise test
Wmean: mean power output during the 10 s sprints
w.w.: wet weight
TAI: Total activation index.
